# Work-Related Musculoskeletal Complaints in Surgeons

**DOI:** 10.3390/healthcare9111482

**Published:** 2021-10-31

**Authors:** Andreea Luciana Rață, Sorin Barac, Loredana Luciana Garleanu, Roxana Ramona Onofrei

**Affiliations:** 1 Department of Vascular Surgery, “Victor Babes” University of Medicine and Pharmacy, Eftimie Murgu Sq no 2, 300042 Timisoara, Romania; rataandreealuciana@gmail.com; 2 “Pius Brinzeu” Emergency County Hospital, 300723 Timisoara, Romania; 3 Emergency City Hospital, 300079 Timisoara, Romania; loredana.garleanu@gmail.com; 4 Department of Rehabilitation, Physical Medicine and Rheumatology, Research Center for Assessment of Human Motion, Functionality and Disability, “Victor Babes” University of Medicine and Pharmacy, 300041 Timisoara, Romania; onofrei.roxana@umft.ro

**Keywords:** musculoskeletal complaints, pain, surgeons

## Abstract

The aim of the present study was to examine the prevalence of work-related musculoskeletal complaints and potential risk factors among Romanian surgeons. Ninety-five surgeons of different specialties (62.11% males) completed a questionnaire about work-related musculoskeletal complaints (WMSCs). Ninety-one surgeons (95.78%) experienced WMSCs at least in one body part in the last year. Most surgeons reported pain in four body parts (33.68%). The most common WMSCs were reported on the lower back (74.73%), followed by complaints in the neck region (55.79%), shoulder and upper back (46.32%), knee (31.58%), wrist–hand (16.84%), elbow (14.74%), hip (11.58%) and ankle–foot (4.21%). Surgeons rated their pain more severe on upper back, lower back and knees. A higher percentage of male surgeons reported upper back pain (χ^2^_(1)_ = 5.818, *p* = 0.015). Significant age differences were found between the reported pain sites (F_8,278_ = 2.666, *p* = 0.008); the surgeons reporting wrist–hand pain were younger than those reporting neck, shoulders, elbows, dorsal and lumbar pain. Surgeons with significantly less experience in years reported significantly more WMSCs in wrist–hand, hip and ankle–foot regions compared with those more experienced (*p* < 0.05). Surgeons are at high risk of developing work-related musculoskeletal complaints, which affects both their professional and personal life. Further studies are needed to identify all risk factors and ergonomic strategies to reduce the prevalence and the negative impact of WMSCs.

## 1. Introduction

Surgeons, as all other healthcare workers, are at risk of developing work-related musculoskeletal complaints (WMSCs) and disorders. A substantial number of surgeons suffer from work-related musculoskeletal symptoms that are exacerbated as those surgeons continue to operate [1].

Several studies have reported that surgeons are exposed to intense physical strain while performing different surgical procedures. All well-known risk factors for WMSCs are met during surgical procedures—awkward, prolonged static postures, repetitive movements of upper limbs [2]. Kant et al. [3] have analyzed the most common static working position adopted by surgeons and this implies the head bent forward, the spine bent forward and twisted, the shoulder raised and standing on one leg. Ruitenberg et al. [4] found that surgeons stand 130% longer and performed fine repetitive movements 26 times longer than other hospital physicians. The physical demands were perceived by surgeons as uncomfortable and exhausting, the main reason being the prolonged repetitive movements and the working postures. The authors concluded that the physical demands of performing surgery are a threat to surgeons’ physical health, work ability and job performance [4]. Another study also described that the discomfort or symptoms reported by surgeons were attributed to performing any mode of surgery—open, laparoscopic or robotic [5].

Yang et al. [6] have analyzed the posture, fatigue and pain across different surgical specialties and procedures. They found that the work posture of surgeons performing open operations was more demanding for neck, trunk and right upper limb compared with other procedures (laparoscopic, endovascular), as measured objectively by a IMU system (inertial measurement units). Of note, 50% of the surgeons participating in that study reported moderate or higher levels of fatigue and clinically significantly neck and lower back pain immediately after the operation. Other studies have also studied the ergonomic risks the surgeons are exposed to during surgeries, using objective ergonomic assessment tools [7,8,9].

WMSCs have a negative impact on surgeons’ professional activities, quality or performance of surgical care. One-third of the surgeons having a physical complaint in the arm or knee felt impaired in their work functioning, and one out of seven surgeons had difficulties coping with their work physical demands due to impairments in their physical well-being [4]. Another study revealed that more than half of the injured surgeons reported at least a minimal impact on their operating performance while recovering from injury [10].

The aim of the present study was to examine the prevalence of work-related musculoskeletal complaints and potential risk factors among Romanian surgeons.

## 2. Materials and Methods

### 2.1. Participants

A total of 95 surgeons of different specialties completed an anonymous questionnaire focused on musculoskeletal complaints. Eligibility criteria were: (1) surgical experience of at least 1 year; (2) no clinically diagnosed inflammatory musculoskeletal disorders; (3) no history of surgeries in the last 6 months.

This cross-sectional study was carried out in accordance with the Declaration of Helsinki and has been approved by the local Ethics (approval No. 173/03.10.2019). Participation in the study was voluntary. Participants who agreed to participate in the study and met the inclusion criteria read and signed an informed consent.

### 2.2. Assessments

The questionnaire was structured in two parts. The first part included questions regarding age, sex, height, weight, working experience, weekly working hours, operating hours per week, number of surgeries/week. The second part included questions about WMSCs, questions that were adapted from the Nordic Musculoskeletal Questionnaire, a validated, repeatable sensitive and useful screening tool [11]. A body diagram was used to help the respondents indicate the affected body parts. If a subject answered with “yes” on the question “Have you had pain in your neck/shoulder/elbow/wrist–hand/upper back/lower back/hip/knee/ankle–foot in the last 12 months?”, then the participant had to answer the questions regarding the pain severity on a 10-point visual analogue scale (VAS), duration, treatment, and the impact of pain on daily living and on professional life.

### 2.3. Statistical Analysis

Data were analyzed with MedCalc Statistical software version 19.2.1 (MedCalc Software Ltd., Ostend, Belgium). Descriptive statistics (means and standard deviation, median and interquartile range (IQR)), number and percentage) were calculated. The relationship between WMSCs and sex, years of experience, daily working hours and number of treated patients was evaluated using Chi-square and Cochrane’s Q tests. In order to compare variables based on the affected regions, a one-way ANOVA with Tukey–Kramer post hoc tests and Kruskal–Wallis with Conover post hoc tests were performed. A stepwise logistic regression was performed in order to identify risk factors for pain in each region. Variables with *p*-values < 0.1 in the univariate logistic analysis were entered in the multivariate logistic analysis. The significance level was set at *p* < 0.05.

## 3. Results

Ninety-five surgeons (62.11% males; height 173.1 ± 8.3 cm; weight 76.75 ± 17.99 kg) agreed to participate and completed the questionnaires. Their age ranged between 25 and 64 years with a mean age of 37.56 ± 8.74 years. Participants’ characteristics are presented in Table 1. The participants had different surgical specialty—general surgery (*n* = 6, 6.32%), vascular surgery (*n* = 10, 10.53%), plastic surgery (*n* = 14, 14.74%), neurosurgery (*n* = 21, 22.11%), orthopedic surgery (*n* = 12, 12.63%), urologic surgery (*n* = 8, 8.42%), cardiac surgery (*n* = 8, 8.42%), thoracic surgery (*n* = 8, 8.42%), obstetric/gynecologic surgery (*n* = 8, 8.42%).

Ninety-one surgeons (95.78%) experienced WMSCs at least in one body part in the last year. Most surgeons reported pain in 4 body parts (*n* = 32, 33.68%), 19 surgeons (20%) in one region, 14 surgeons (14.74%) in 2 regions, 13 surgeons (13.68%) in 5 regions, 11 (11.58%) in 3 regions and only 2 surgeons (2.1%) in 6 regions. The most common WMSCs were reported on the lower back (*n* = 71, 74.73%), followed by complaints in the neck region (*n* = 53, 55.79%), shoulder and upper back (*n* = 44, 46.32%), knee (*n* = 30, 31.58%), wrist–hand (*n* = 16, 16.84%), elbow (*n* = 14, 14.74%), hip (*n* = 11, 11.58%) and ankle–foot (*n* = 4, 4.21%). Pain and subjects’ characteristics based on the affected region are presented in Table 2.

Gender differences were found only for upper back pain, with a higher percentage of male surgeons reporting WMSCs in this area (χ^2^_(1)_ = 5.818, *p* = 0.015). Significant age differences were found between the reported pain sites (F_8,278_ = 2.666, *p* = 0.008); the surgeons reporting wrist–hand pain were younger elbow pain (*p* < 0.05).

Surgical experience was significantly correlated with neck pain (χ^2^_(5)_ = 43.11, *p* < 0.0001), shoulder pain (χ^2^_(3)_ = 16.36, *p* = 0.001), upper back pain (χ^2^_(5)_ = 20.36, *p* = 0.001), lower back pain (χ^2^_(5)_ = 42.83, *p* < 0.0001) and knee pain (χ^2^_(3)_ = 11.33, *p* = 0.01), with those with less than 10 years of experience reporting more WMSCs. Surgeons with significantly less experience years reported significantly more WMSCs in wrist–hand, hip and ankle–foot regions compared with those more experienced (*p* < 0.05).

Surgeons with shoulder pain reported significantly more operating hours/week than those with pain in the cervical, wrist–hand, upper back and ankle–foot regions (*p* < 0.05).

The analysis of the prevalence of WMSCs by surgical specialties revealed that the elbow, upper back and lower back pain was highest in neurosurgeons, while upper and lower back were also prevalent in plastic surgeons (Table 3).

Data related to pain and its impact on surgeons’ activities are presented in Table 4. Pain severity differed significantly across regions (F_8,287_ = 37.77, *p* < 0.0001). Surgeons rated their pain more severe on upper back, lower back and knees compared with all other sites (*p* < 0.05).

A significantly number of surgeons reported pain in the neck and lower back for more than 30 days in the last 12 months (cervical pain χ^2^_(2)_ = 18.42, *p* < 0.001; lower back χ^2^_(2)_ = 29.18, *p* < 0.001). In regards to shoulder, elbow and wrist–hand regions, significantly more surgeons reported pain with a duration of less than 7 days (shoulder χ^2^_(2)_ = 9.86, *p* = 0.007; elbow χ^2^_(2)_ = 17.71, *p* < 0.001); wrist–hand χ^2^_(2)_ = 26.37, *p* < 0.001).

The impact of pain on professional activities, leisure activities or both differed significantly according to the affected region (χ^2^_(8)_ = 30.65, *p* < 0.001; χ^2^_(8)_ = 55.59, *p* < 0.001; χ^2^_(8)_ = 125.73, *p* < 0.001, respectively). The number of surgeons with neck pain whose professional activities were affected by WMSCs was significantly higher compared with those with elbow, wrist–hand and ankle–foot pain (*p* < 0.05). Leisure activities and both professional and leisure activities were affected in a higher proportion by lower back pain compared to the other affected regions (*p* < 0.05). Neck and knee pain affected both professional and leisure activities in a higher percentage than elbow, hip and ankle–foot pain (*p* < 0.05). The majority of surgeons continued working with pain.

Significant differences were observed in the number of surgeons receiving medical treatment for their WMSCs (χ^2^_(8)_ = 73.78, *p* < 0.001), a higher prevalence of surgeons with lower back pain following medical treatment in the last 12 months compared with those with pain located in other body parts (*p* < 0.05). Four surgeons with lower back pain (5.63%) needed sick leave due to their musculoskeletal complaints.

The logistic regression analysis revealed that a higher BMI was a risk factor for upper back pain (OR-1.1, 95% CI: 1–1.21, *p* = 0.04) and elbow pain (OR-1.14, 95% CI: 1.02–1.27, *p* = 0.01). Being female increased the risk for neck pain (OR-2.63, 95% CI: 1.07–6.44, *p* = 0.03). A sedentary lifestyle proved to be a risk factor for shoulder pain (OR-8.7, 95% CI: 2.99-25.29, *p* = 0.0001) and for elbow pain (OR-5.53, 95% CI: 1.5–20.37, *p* = 0.01). Other risk factors related to professional activities were the number of surgeries performed in the last 6 months for neck pain (OR-1.13, 95% CI: 1.01–1.27, *p* = 0.03) and shoulder pain (OR-1.29, 95% CI: 1.12–1.49, *p* = 0.0003); number of weekly working hours for lower back pain (OR-1.13, 95% CI: 1.04–1.21, *p* = 0.001) and for hip pain (OR-1.09, 95% CI: 1.03–1.15, *p* = 0.002); wearing a lead apron during surgeries for upper back pain (OR-3.66, 95% CI: 1.32–10.08, *p* = 0.01).

## 4. Discussion

The aim of the present study was to examine the prevalence of work-related musculoskeletal complaints and potential risk factors among Romanian surgeons. The main finding of this study was that the surgeons are at high risk for development of work-related musculoskeletal complaints; 95.78% of the respondent surgeons have experienced WMSCs in at least one body part in the last 12 months. Most surgeons reported pain in 4 body parts (33.68%).

Our findings are in agreement with previous results, suggesting that surgeons are at high risk for developing WMSCs [12,13,14,15,16,17,18]. Alnefaie et al. [12] reported that 80% of respondent suffered from musculoskeletal manifestations related to surgery, with back and neck being the most affected parts (71.1% and 59.8%, respectively). Dianat et al. [14] found a prevalence of 77.2% of surgeons reporting musculoskeletal symptoms, with 76% of these with pain or discomfort in more than one body region. In the study of Szeto et al. [13], over 80% respondent surgeons reported experiencing at least one area of musculoskeletal symptoms in the past 12 months, neck region (82.9%) being the most affected, followed by lower back (68.1%), shoulder (57.8%) and upper back (52.6%).

The most affected region in our study was lower back (74.73%), followed by complaints in the neck region (55.79%), shoulder and upper back (each 46.32%), knee (31.58%), wrist–hand (16.84%), elbow (14.74%), hip (11.58%) and ankle–foot (4.21%). Similar results were also reported by Auerbach et al. [19], who identified a prevalence of lower back pain of 62% and neck pain of 59%. Radiculopathy was present in 30% cases with lower back pain and 28% cases with neck pain. Upper limb complaints were also prevalent in their study sample, 49% reporting shoulder pain and 24% rotator cuff symptoms. Other studies reported the neck as the most affected region [2,13,15,16,18]. In a study of 141 surgeons of different specialties, Giagio et al. [2] reported the most frequently affected body regions as being the neck (79%), lower back (75%), upper back (59%), shoulders (51%), and wrist and hand (26%). In the study of Kokosis et al. [16], 94% of the responders (plastic surgeons) have experienced musculoskeletal pain, with neck being the most affected area.

In our study, surgeons reported more intense pain at the upper back, lower back and knee, with a higher number of surgeons experiencing pain for more than 30 days in the last 12 months. Lower back pain had a significant impact on both leisure and professional activities, with 39.44% of surgeons receiving medical treatment to ameliorate WMSCs in the lower back. In all cases, the surgeons continued working with pain. Our findings are in accordance with those published by Dianat et al. [14], who also reported the mean severity rating of the symptoms experienced by the surgeon being moderate and high. In their study, almost half of the surgeons reported disruption of their normal activities due to musculoskeletal symptoms. Davis et al. [10] reported that 40% of the surgeons included in their study sustained a musculoskeletal injury in the workplace during their career, the common injured regions being the back, hand and neck. In half of the cases, the pain lasted more than 1 month, 66% of injuries being attributed to chronic causes as strain from the operating posture.

The high prevalence of musculoskeletal complaints among surgeons could be attributed to the physical demands of prolonged static working positions and postures, repetitive movements of the upper limbs during surgeries, with very fine eye–hand coordination, long working hours [2,5,13,17]. Yang et al. reported that the working posture of surgeons performing open surgeries was demanding for the neck, trunk and right upper extremity [6]. Not only the bad posture, but also the equipment use during surgery could be considered a cause of musculoskeletal complaints [18]. Our results showed that the majority of surgeons reporting WMSCs wore a lead apron during surgery.

We found gender differences only for upper back pain, with a higher percentage of male surgeons reporting WMSCs in this area. Previous studies showed a higher prevalence of symptoms in female surgeons in the neck, shoulders, elbows, hand/wrists, upper back, hips, knees and ankles [14]. Female surgeons have been reported to experience more pain and discomfort in the wrists [18] and to be at higher risk for multisite musculoskeletal pain than male surgeons [20]. In our study, being female proved to increase the risk for neck pain, maybe as a result of an ergonomic disadvantage of being shorter. Sutton et al. [21] considered that the higher prevalence of symptoms reported by female surgeons in the shoulder area is due to the fact that female surgeons need to accommodate to the operating table height by raising their arms. Similar results were also reported by Adams et al. [22], who hypothesized that being shorter and having less upper body strength place female surgeons at risk of developing musculoskeletal symptoms or disorders.

Our results revealed that wrist–hand pain was more frequent in younger surgeons. Moreover, surgeons with less years of experience experienced more WMSCs in the wrist–hand, hip and ankle–foot regions compared with those more experienced. Neck, shoulder, upper and lower back and knee pain were more frequent in those with less than 10 years of experience. Similar results were reported by Alnefaie et al. [12], who found a higher percentage of surgeons with 5–10 years of practice with musculoskeletal manifestations, and also by Kokosis et al. [16], who found that musculoskeletal complaints started early in the training of plastic surgeons. Mavrovounis et al. [23] specified that musculoskeletal symptoms appeared early in the residency in their study sample of neurosurgeons. Hemal et al. [24] found that finger numbness is more common in junior laparoscopic surgeons than in senior surgeons. This aspect could be explained by the limited experience, which could lead to inefficient practice. Early training regarding surgery ergonomics could be of much help in preventing musculoskeletal complaints in surgeons. Giagio et al. [2] found that career longevity of more than 20 years is a protective factor for WMSCs. On the other hand, it might be expected that older surgeons, with many years of experience, would be at risk for developing musculoskeletal complaints, as a result of cumulative exposure to physical demands and stress during surgeries.

The analysis of the prevalence of WMSCs by surgical specialties revealed that the elbow, upper back and lower back pain was highest in neurosurgeons, while upper and lower back were more frequent also in plastic surgeons.

A higher BMI and a sedentary lifestyle proved to increase the risk of upper back, shoulder and elbow pain. Dianat et al. [14] reported that the prevalence of neck, shoulder and lower back symptoms decreased with more time spent on sport/physical activities.

Our findings are in agreement with other studies which showed that the professional high physical demand (number of surgeries, number of weekly working hours) increases the risk of work-related musculoskeletal complaints [4,13,14,20].

Due to the high frequency of work-related musculoskeletal complaints and the impact on surgeons’ professional and personal life, we recommend preventive programs that will raise awareness of the importance of ergonomics, working postures, surgery schedules and active breaks.

The present study has some limitations. The relatively small sample size, not including all surgical specialties, and the retrospective recall of symptoms are some of the limitations. No objective assessment tool was used to identify the musculoskeletal complaints. We have not considered the working positions, and this is an aspect that should be considered in future studies in order to prevent work-related musculoskeletal complaints due to bad posture and positions during surgeries. Another limitation is that we did not study the loupes usage, which can lead to supplementary load of the neck and, consequently, higher pain in that region.

## 5. Conclusions

Romanian surgeons are at high risk for work-related musculoskeletal complaints that affect both professional and personal life. Further studies are needed to identify all risk factors and ergonomic and educational strategies to reduce the prevalence and the negative impact of WMSCs.

## Figures and Tables

**Table 1:** Participants’ characteristics.

Variable	
Age, years (mean ± SD)	37.56 ± 8.74
Sex	
male, *n* (%)	59 (62.11)
female, *n* (%)	36 (37.89)
Weight, kg (mean ± SD)	76.75 ± 17.99
Height, cm (mean ± SD)	173.1 ± 8.3
BMI, kg/m^2^ (mean ± SD)	25.44 ± 5.04
Dominant hand	
Right, *n* (%)	89 (93.68)
Left, *n* (%)	6 (6.32)
Physical activities, *n* (%)	60 (63.16)
Number of days performing physical activities (median (IQR))	2 [0–4]
Sleeping time, hours/day (mean ± SD)	6.72 ± 0.96
Surgical experience, years (mean ± SD)	10.09 ± 8.41
Weekly working hours (mean ± SD)	41.03 ± 14.62
Surgery hours/week (mean ± SD)	13.07 ± 7.27
Number of surgeries/week in the last 6 months (mean ± SD)	7.56 ± 3.73
Wearing lead apron, *n* (%)	67 (70.53)
Number of hours with lead apron/day (median (IQR))	1 [0–2]

BMI—body mass index.

**Table 2:** Subjects’ characteristics related to the affected region.

Parameters	Neck Pain (*n* = 53)	Shoulder Pain (*n* = 44)	Elbow Pain (*n* = 14)	Wrist–Hand Pain (*n* = 16)	Upper Back Pain (*n* = 44)	Lower Back Pain (*n* = 71)	Hip Pain (*n* = 11)	Knee Pain (*n* = 30)	Ankle–Foot Pain (*n* = 4)
Sex									
male, *n* (%)	28 (52.83)	24 (54.55)	6 (42.86)	6 (37.5)	30 (68.18)	41 (57.75)	6 (54.55)	18 (60)	2 (50)
female, *n* (%)	25 (47.17)	20 (45.45)	8 (57.14)	10 (62.5)	14 (31.82)	30 (42.25)	5 (45.45)	12 (40)	2 (50)
Age, years (mean ± SD)	37.81 ± 10.47	38.95 ± 9.81	42.57 ± 8.54	30.75 ± 2.41	38.59 ± 9.79	38.05 ± 9.42	31.82 ± 5.27	43.5 ± 14.39	30.5 ± 0.58
Height, cm (mean ± SD)	172.86 ± 8.35	172.06 ± 7.68	169 ± 4.54	172.32 ± 12.24	174.45 ± 7.11	172.9 ± 8.19	176.09 ± 6.71	173.13 ± 8.68	168 ± 13.85
Weight, kg (mean ± SD)	77.79 ± 17.63	78.95 ± 22.23	83.38 ± 17.72	69.18 ± 22.55	80.68 ± 19.14	72.46 ± 17.97	68.54 ± 15.62	73.36 ± 14.8	65 ± 18.47
BMI, kg/m^2^ (mean ± SD)	25.92 ± 5.25	26.45 ± 6.49	29.03 ± 5.27	22.82 ± 5.11	26.36 ± 5.6	25.43 ± 5.19	21.87 ± 3.63	24.42 ± 4.42	22.56 ± 2.81
BMI > 25 kg/m^2^, *n* (%)	18 (33.96)	18 (40.91)	10 (71.42)	2 (12.5)	20 (45.45)	25 (35.21)	2 (18.18)	10 (33.33)	0 (0)
Physical activity, *n* (%)	33 (62.26)	17 (38.63)	4 (28.57)	10 (62.5)	26 (59.09)	42 (59.15)	6 (54.54)	15 (50)	4 (100)
Wearing lead apron, *n* (%)	38 (71.7)	28 (63.64)	10 (71.43)	14 (87.5)	36 (81.82)	51 (71.83)	11 (100)	24 (80)	4 (100)
Surgical experience, years (median (IQR))	6 [2–14]	8 [6–14]	9 [6–28]	3 [2.5–6]	8 [3–20]	8 [3–15]	3 [1–8]	8 [3–10]	2 [2–2]
1–5 years, *n* (%)	22 (41.51)	10 (22.73)	2 (14.29)	9 (56.25)	12 (27.27)	29 (40.85)	7 (63.64)	11 (36.67)	4 (100)
6–10 years, *n* (%)	17 (32.08)	22 (50)	6 (42.85)	7 (43.75)	16 (36.36)	19 (26.76)	2 (18.18)	13 (43.33)	0 (0)
11–15 years, *n* (%)	2 (3.77)	4 (9.09)	0 (0)	0 (0)	2 (4.55)	7 (9.86)	0 (0)	0 (0)	0 (0)
16–20 years, *n* (%)	2 (3.77)	0 (0)	2 (14.29)	0 (0)	4 (9.09)	4 (5.63)	2 (18.18)	2 (6.67)	0 (0)
21–25 years, *n* (%)	2 (3.77)	0 (0)	0 (0)	0 (0)	6 (13.64)	4 (5.63)	0 (0)	0 (0)	0 (0)
26–30 years, *n* (%)	8 (15.09)	8 (18.18)	4 (28.57)	0 (0)	4 (9.09)	8 (11.27)	0 (0)	4 (13.33)	0 (0)
Surgery hours/week (median (IQR))	10 (6–16.25)	14.5 (10–20)	10 (6–25)	10 (4.5–20)	10 (8–18)	12 (10–20)	10 (10–18)	10 (4–20)	3 (3–3.5)

**Table 3:** Prevalence of WMSCs by surgical specialty.

Parameters	Neck Pain (*n* = 53)	Shoulder Pain (*n* = 44)	Elbow Pain (*n* = 14)	Wrist–Hand Pain (*n* = 16)	Upper Back Pain (*n* = 44)	Lower Back Pain (*n* = 71)	Hip Pain (*n* = 11)	Knee Pain (*n* = 30)	Ankle–Foot Pain (*n* = 4)
Surgical specialty									
General, *n* (%)	5 (9.43)	2 (4.54)	2 (14.28)	0 (0)	2 (4.54)	6 (8.45)	2 (18.18)	4 (13.33)	0 (0)
Vascular, *n* (%)	3 (5.66)	4 (9.09)	0 (0)	1 (6.25)	8 (18.18)	8 (11.27)	3 (27.28)	1 (3.34)	0 (0)
Plastic, *n* (%)	10 (18.87)	2 (4.54)	0 (0)	4 (25)	10 (22.73)	10 (14.08)	2 (18.18)	4 (13.33)	2 (50)
Neurosurgery, *n* (%)	11 (20.75)	8 (18.18)	10 (71.44)	3 (18.75)	10 (22.73)	19 (26.76)	0 (0)	5 (16.67)	0 (0)
Orthopaedic, *n* (%)	6 (11.32)	8 (18.18)	0 (0)	4 (25)	6 (13.64)	8 (11.27)	2 (18.18)	4 (13.33)	0 (0)
Urologic, *n* (%)	8 (15.09)	4 (9.09)	0 (0)	2 (12.5)	4 (9.09)	2 (2.82)	0 (0)	4 (13.33)	2 (50)
Cardiac, *n* (%)	4 (7.55)	6 (13.64)	0 (0)	0 (0)	2 (4.54)	6 (8.45)	0 (0)	4 (13.33)	0 (0)
Thoracic, *n* (%)	2 (3.77)	2 (4.54)	0 (0)	0 (0)	2 (4.54)	6 (8.45)	0 (0)	0 (0)	0 (0)
Obstetric/gynecologic, *n* (%)	4 (7.55)	8 (18.18)	2 (14.28)	2 (12.5)	0 (0)	6 (8.45)	2 (18.18)	4 (13.33)	0 (0)
*p* *	0.09	0.45	**0.01**	0.73	**0.02**	**0.004**	0.98	0.92	NA

* p—relates to differences between surgical specialties. Bold: the statistical-signifiant values.

**Table 4:** Pain characteristics based on regions.

Parameters	Neck Pain (*n* = 53)	Shoulder Pain (*n* = 44)	Elbow Pain (*n* = 14)	Wrist–Hand Pain (*n* = 16)	Upper Back Pain (*n* = 44)	Lower Back Pain (*n* = 71)	Hip Pain (*n* = 11)	Knee Pain (*n* = 30)	Ankle–Foot Pain (*n* = 4)
VAS (median (IQR))	4 (3–5)	4 (3–5)	4 (2–4)	3.5 (2.5–4)	5 (4–7)	5 (4–7)	4 (3–5)	5 (4–6)	3 (2.5–3)
Pain duration									
1–7 days, *n* (%)	10 (18.87)	23 (52.27)	12 (85.71)	15 (93.75)	14 (31.82)	15 (21.13)	4 (36.36)	8 (26.67)	1 (25)
8–30 days, *n* (%)	15 (28.30)	6 (13.64)	2 (14.29)	0 (0)	14 (31.82)	11 (15.49)	1 (9.09)	7 (23.33)	1 (25)
>30 days, *n* (%)	28 (52.83)	15 (34.09)	0 (0)	1 (6.25)	16 (36.36)	45 (63.38)	6 (54.55)	15 (50)	2 (50)
Impact on professional activity, *n* (%)	16 (30.19)	10 (22.73)	4 (28.57)	4 (25)	8 (18.18)	12 (16.90)	6 (54.55)	8 (26.67)	0 (0)
Impact on leisure activities, *n* (%)	0 (0)	3 (6.82)	0 (0)	0 (0)	0 (0)	4 (5.63)	1 (9.09)	1 (3.33)	0 (0)
Impact on both professional and leisure activities, *n* (%)	20 (37.73)	11 (25)	0 (0)	8 (50)	8 (18.18)	41 (57.75)	2 (18.18)	19 (63.33)	4 (100)
Continue working with pain, *n* (%)	41 (77.36)	34 (77.27)	12 (85.71)	16 (100)	38 (86.36)	54 (76.05)	7 (63.64)	22 (73.33)	4 (100)
Received medical treatment, *n* (%)	4 (7.54)	16 (36.36)	4 (28.57)	2 (12.5)	8 (18.18)	28 (39.44)	3 (27.27)	12 (40)	0 (0)
Sick leave, *n* (%)	0 (0)	0 (0)	0 (0)	0 (0)	0 (0)	4 (5.63)	0 (0)	5 (16.67)	0 (0)

## Data Availability

Not applicable.
